# Viral metagenomes of Lake Soyang, the largest freshwater lake in South Korea

**DOI:** 10.1038/s41597-020-00695-9

**Published:** 2020-10-13

**Authors:** Kira Moon, Suhyun Kim, Ilnam Kang, Jang-Cheon Cho

**Affiliations:** 1 Department of Biological Sciences, Inha University, Incheon, 22212 Republic of Korea; 2 Center for Molecular and Cell Biology, Inha University, Incheon, 22212 Republic of Korea

**Keywords:** Microbial ecology, Bacteriophages

## Abstract

A high number of viral metagenomes have revealed countless genomes of putative bacteriophages that have not yet been identified due to limitations in bacteriophage cultures. However, most virome studies have been focused on marine or gut environments, thereby leaving the viral community structure of freshwater lakes unclear. Because the lakes located around the globe have independent ecosystems with unique characteristics, viral community structures are also distinctive but comparable. Here, we present data on viral metagenomes that were seasonally collected at a depth of 1 m from Lake Soyang, the largest freshwater reservoir in South Korea. Through shotgun metagenome sequencing using the Illumina MiSeq platform, 3.08 to 5.54-Gbps of reads per virome were obtained. To predict the viral genome sequences within Lake Soyang, contigs were constructed and 648 to 1,004 putative viral contigs were obtained per sample. We expect that both viral metagenome reads and viral contigs would contribute in comparing and understanding of viral communities among different freshwater lakes depending on seasonal changes.

## Abstract (summary)

**Table:** 

Measurement(s)	Metagenome • DNA viral genome
Technology Type(s)	whole genome sequencing
Factor Type(s)	season
Sample Characteristic - Organism	unclassified bacterial viruses
Sample Characteristic - Environment	oligotrophic lake • freshwater lake biome
Sample Characteristic - Location	South Korea

Machine-accessible metadata file describing the reported data: 10.6084/m9.figshare.12924506

## Background & Summary

Bacteriophages—viruses that infect bacteria—are the smallest but the most abundantly found biological entities on earth with approximately 10^31^ particles^1^. Despite their large population, only about 2,500 bacteriophages (phage) genomes have been announced so far (as of April 2020, RefSeq release 99, www.vogdb.org). A low number of phage isolates have been reported owing to difficulties in isolating and culturing phages in laboratory settings. As obligate parasites of bacteria, phage cultivation requires the preceding culture of bacterial hosts. However, most of the bacterial population remains uncultured^2^ despite the development of various culturing techniques. Consequently, phages are left as a large black box of the biosphere. Advances in viral metagenome (virome) studies have facilitated access to a vast amount of phage genomes without the need for phage cultivation. Although most of the virome sequences remain uninterpretable in terms of viral taxonomy and their host information due to a dearth of reference phage genomes across public databases, viral community structures within diverse environments, including ocean^3^, freshwater^4^, soil^5^, and gut^6^, have been accessed and predicted. The virome sequences are valuable for quantifying the abundance of specific phage genomes within the environment *in silico*^3^. The virome data can also be used to predict putative host-phage systems by matching virome sequences to the CRISPR array sequences of bacterial CRISPR-Cas systems^7,8^ or bacterial signature genes^9,10^. Virome sequences are also very useful for the discovery of novel phage genes, including antibiotic resistance genes, that are carried by uncultured phages^11,12^.

Most of the virome studies have been focused on marine and gut environments, leaving freshwater viral communities in question^1,8^. As one of the major biospheres of earth, freshwater systems encompass diverse organisms, including methylotrophic, nitrifying, and sulfur-oxidizing bacteria, that contribute to biogeochemical cycles. Hence, numerous ecological and genomic studies on freshwater bacterial communities have been performed, whereas a lesser number of studies have been conducted on phages. Lake Soyang is the largest artificial freshwater reservoir in South Korea that is represented in GLEON (Global Lake Ecological Observatory Network^13^). As a temperate monomictic lake with seasonal physicochemical turnovers and phytoplankton blooms^14^, Lake Soyang is rich in microbial diversity. From Lake Soyang, numerous novel bacterial strains and phages have been isolated and cultured^15–18^. Particularly, phage P19250A, which is the most abundantly found freshwater phage that infects the methylotrophic bacterial strain “*Candidatus* Methylopumilus planktonicus,” was originally isolated and cultured from Lake Soyang^19^. Novel phages, P26218^16^ and P26059A, and B^15^ infecting heterotrophic bacterial strains isolated from Lake Soyang such as *Rhodoferax lacus*^17^ and a bacterial strain belonging to the *Comamonadaceae* family, respectively, have also been isolated from the Lake Soyang. Bacterial strains from the acI group of the *Actinobacteria* phylum, which is a ubiquitous and the most frequently found freshwater bacterial group, has been recently isolated and successfully cultured from Lake Soyang for the very first time^20^.

**Fig. 1:**
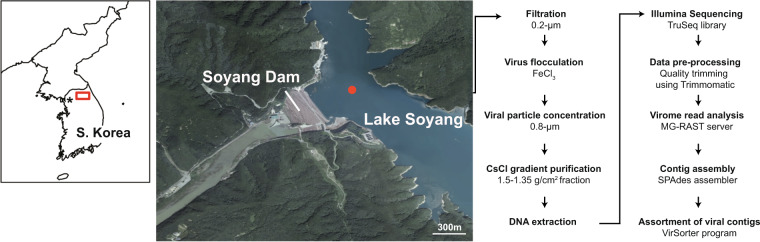
A map depicting the sampling site in Lake Soyang and an overview of the metagenome preparation. The red dot represents the sampling site.

**Fig. 2:**
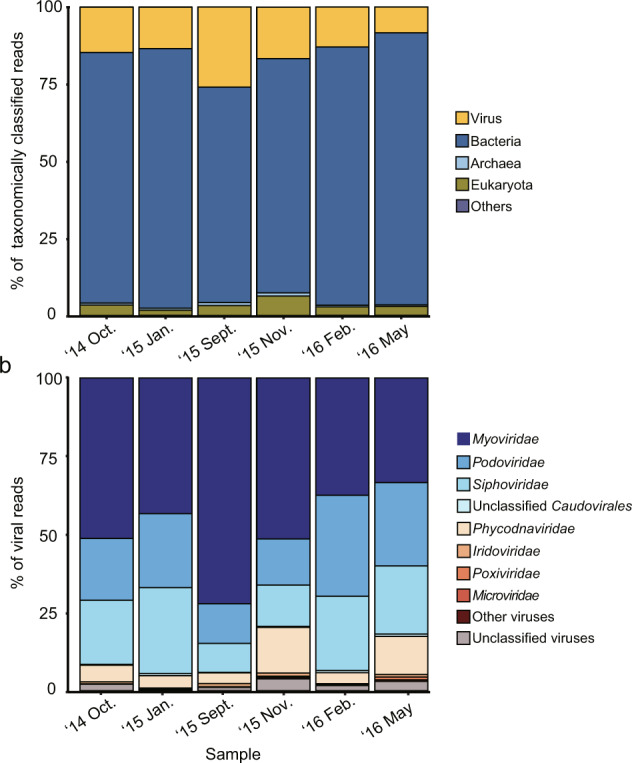
Taxonomic annotation of virome reads collected from Lake Soyang. The taxonomic prediction of virome reads is shown in the domain level (**a**). Only the virome reads that were able to be taxonomically classified by MG-RAST using the NCBI RefSeq database are shown here. The “others” shown here means reads that had a significant hit in RefSeq database but could not be assigned to a specific taxon. The reads that were annotated as viruses in (**a**) were further shown in family levels in (**b**).

Here, we present six viral metagenomes collected from the surface of Lake Soyang from October 2014 to May 2016. Each virome represents different seasons and is expected to show a seasonal shift in viral community structures as late-autumn turnover takes place in the monomictic lake^14^. The collected water samples were enriched for virus particles using a combination of filtration, precipitation, and CsCl purification targeting double-stranded DNA phages (see Fig. 1 & Methods). The viromes were sequenced with Illumina MiSeq platform, and each virome yielded between approximately 3.08-Gbps to 5.54-Gbps of raw sequencing reads (Table 1). The proportion of bacterial rRNA sequences and marker genes was low, and viral gene enrichment scores were approximately 3 to 8-fold higher compared with non-viral metagenome data, showing that the possibility of bacterial contamination in the virome reads was negligible (Table 1). The virome reads were subjected for taxonomic prediction via MG-RAST server (mg-rast.org)^21^, with 3.1 to 17.3% of the reads taxonomically classified. Of those classified reads, 8.4 to 26.0% were predicted to be of viral origin (Fig. 2a) and most of the reads assigned to viruses belonged to the order *Caudovirales* (Fig. 2b). The virome contigs were assembled from the virome reads, resulting in a total of 809,964 contigs with minimum length of 128 bp; 6,480 of these contigs with a sequence length longer than 10-kb were used for further analysis (Table 2). Among those, 5,203 contigs were predicted as either viral or prophage genomes (Table 2), according to VirSorter^22^. A low proportion of bacterial sequences and a high proportion of viral contigs in the virome data of Lake Soyang indicate that the viral community sequences were well sampled. Therefore, we anticipate that the virome data of Lake Soyang would prove to be a useful resource for facilitating the discovery of novel phage genomes and the study of seasonal changes in viral community structure.

**Table 1:** Sequencing information of viral metagenomes from Lake Soyang.

Sample	Accession no.	Base pair (Gbp)	% of SSU rRNA^a^	% of LSU rRNA^a^	% of Bacterial markers^a^	Score^a^
′14 Oct.	ERR2814725	3.20	0.0013	0.0192	0.0195	6.88079
′15 Jan.	ERR2814726	3.08	0.0004	0.0161	0.0074	8.23119
′15 Sept.	ERR2814753	5.54	0.0017	0.0330	0.0079	3.99835
′15 Nov.	ERR2814752	3.69	0.0040	0.0212	0.0440	6.23944
′16 Feb.	ERR2814750	3.25	0.0031	0.0159	0.0146	8.29454
′16 May	ERR2814751	3.18	0.0091	0.0047	0.0957	2.95373

^a^The degree of bacterial gene contamination, as determined by the ratio of bacterial marker genes, was calculated using the ViromeQC program^28^.

**Table 2:** Number of virome contigs assembled from Lake Soyang virome reads.

Sample	IMG Accession no.	Assembled contigs	N50 (bp)	Assembled total bases	Length of longest contigs	Contigs ≥ 10-kb	Viral contigs^a^
′14 Oct.	3300007735	78,169	1,950	23,735,463	213,274	1,027	867
′15 Jan.	3300007734	89,763	1,577	22,031,041	176,311	983	844
′15 Sept.	3300011113	121,633	1,324	19,395,483	334,901	835	648
′15 Nov.	3300011116	214,755	1,084	30,660,637	334,837	1,352	1,004
′16 Feb.	3300011114	164,680	1,071	22,677,118	125,970	1,112	935
′16 May	3300011115	140,964	1,266	24,544,035	215,674	1,171	867

^a^The number of viral and prophage contigs were determined using the VirSorter program^22^.

## Methods

### Environmental Sampling and metagenome sequencing

From October 2014 to May 2016, 20 L of water samples were collected six times at a depth of 1 m from the Dam station of Lake Soyang, located in Gangwon province, South Korea (37.947421 N, 127.818872 E, Fig. 1). Physicochemical parameters such as temperature, concentration of dissolved oxygen (DO), and pH were measured on site using the YSI Multi-parameter water quality meter, 556 MPS model (Table 3, YSI Incorporated, Yellow Springs, OH, USA). The other physicochemical parameters were measured using the HACH spectrophotometer (HACH DR-28000, Loveland, CA, USA) or QuAAtro microflow analyzer (SEAL analytical, Mequon, Wisconsin, USA). Using the HACH spectrophotometer, 14’ Oct. and ’15 Jan. samples were analyzed for ammonia (HACH method 8155), nitrite (HACH method 8507), nitrate (HACH method 8171), phosphorous (HACH method 8048), and silica (HACH method 8186), according to the manufacturer’s instructions. The collected water samples were maintained at 4 °C and brought to the laboratory. Upon arrival at the lab, 5 L of each water sample was filtered through a 142 mm 0.2-μm Supor® PES Membrane filter (Pall Corporation, New York, USA) using a polycarbonate filter holder (Geotech, Denver, CO, USA) to remove bacteria-like particles. Five milligrams of FeCl_3_·6H_2_O was added to 5 L of filtered water samples for flocculating viral particles within the samples^23^. The samples were incubated at room temperature for 1 hour, with intermittent vigorous shaking to promote flocculation of viral particles. The flocculated viral particles were then collected on a 0.8-μm Isopore polycarbonate filter (Merck Millipore, Darmstadt, Germany). The polycarbonate filters were placed in a conical tube and stored at 4 °C under dark conditions with moist until further treatment^24^.

**Table 3:** Physicochemical features of Lake Soyang water samples.

Sample	Temp.(°C)^a^	Salinity (%)^a^	DO (mg/L)^a^	pH^a^	PO_4_^3−^ (mg/L)^b^	SiO_2_ (mg/L)^b^	NH_3_-N (mg/L)^b^	NO_2_^–^N (mg/L)^b^	NO_3_^–^N (mg/L)^b^
′14 Oct.^c^	19.49	0.00	8.49	6.18	0.0100	2.372	0.0100	0.0070	1.900
′15 Jan.^c^	5.56	0.04	6.07	6.89	0.0100	2.614	0.0000	0.0060	1.800
′15 Sept.^d^	25.64	0.05	8.29	8.43	ND^f^	1.5241	0.0337	0.0195	1.5331
′15 Nov.^d^	16.55	0.04	6.92	7.88	ND^f^	0.8486	0.0267	0.0024	1.6485
′16 Feb.^d^	4.97	0.15	7.54	7.42	0.0009	1.0927	0.0088	0.0014	1.5802
′16 May^d^	14.01	0.06	NA^e^	7.02	ND^f^	2.2380	0.0167	0.0091	1.4776

^a^The physical measurements of the water samples were measured and recorded on site using the YSI 556 MPS instrument.

^b^The physicochemical values were measured in laboratory setting using either HACH spectrophotometer instrument or QuAAtro microflow analyzer.

^c^The physicochemical values for these samples were measured using the HACH spectrophotometer instruments.

^d^The physicochemical values for these samples were measured using the QuAAtro microflow analyzer.

^e^Not available.

^f^Not detected.

The polycarbonate filters were inoculated in 5 ml of 0.1 M EDTA-0.2 M MgCl_2_-0.2 M ascorbate acid buffer (pH 6) to resuspend flocculated viral particles. Then, the resuspended viral concentrate was treated with DNase I and RNase A at final concentrations of 10 U/ml and 1 U/ml (Sigma-Aldrich, St. Louis, MO, USA), respectively, to remove external nucleic acids. After incubating for 1 hour of incubation with both enzymes at 20 °C, DNase and RNase were deactivated by adding EDTA and EGTA^25^ at final concentrations of 100 mM. The nuclease-treated viral particles were purified via cesium chloride (CsCl) step-gradient ultracentrifugation^26^. Different densities of CsCl were stacked from bottom to top layers in a centrifuge tube in the following order: 1.7, 1.5, 1.35, and 1.2 g/cm^3^; above the top layer, approximately 15 ml of viral particles was added. The samples were centrifuged at 24,000 rpm for 4 hours at 4 °C in a Beckman Coulter L-90K ultracentrifuge with an SW32 Ti swing bucket. After centrifugation, the density fraction ranging between 1.5 and 1.35 g/cm^3^, corresponding to the density of double-stranded DNA phages, especially of *Caudovirales*, was retrieved using a syringe. The CsCl remnants in the sample were removed through washing with SM buffer (50 mM Tris-HCl, pH 7.5; 100 mM NaCl; 10 mM MgSO_4_·7H_2_O; 0.01% gelatin). The samples were loaded onto the 30 kDa Centrifugal Device (Pall Corporation) and centrifuged at 3,000 rpm until the supernatants were flowed through. Then 10 ml of SM buffer was added to resuspend the sample and was centrifuged again. This process was repeated three times to wash out the CsCl. To remove any remaining bacterial-size contaminants, the samples were filtered through a 0.2-μm pore size Acrodisc® Syringe filter with Supor® membrane (Pall Corporation). Viral DNA was extracted from the filtrates using the Qiagen DNeasy Blood and Tissue Kit (Qiagen, Hilden, Germany), according to the manufacturer’s instruction with a slight modification^24^. To 70 μl of the sample, 300 μl of ATL buffer, 30 μl of Proteinase K, and 6 μl of RNase A were added to lyse capsid proteins, followed by addition of 300 μl of 99% ethanol and AL buffer. Then DNA was washed and eluted using the spin column. The extracted viral DNA (211–702 ng per each sample) was subsequently used for constructing a TruSeq library without any amplification. Sequencing was performed at ChunLab Inc. (Seoul, South Korea) using Illumina MiSeq platform, with 2 × 300-bp paired-end reads and no sequencing controls was used. The overall schematic for viral metagenome preparation is shown in Fig. 1.

### Quality trimming, assembly, and analysis of viral metagenome reads and contigs

Using the CLC Genomics Workbench (Qiagen), the raw metagenome sequencing data were mapped to the phiX174 genome to remove technical sequencing control reads. The virome data were uploaded in the MG-RAST server^21^. Taxonomic assignment of the virome reads were performed with the analysis tools provided by the MG-RAST, using the RefSeq as a reference database with default parameters (Fig. 2). The MG-RAST pipeline predicts potential protein encoding genes from each read and compare these translated sequences against reference databases for taxonomic and functional assignment^21^.

The virome reads with phiX174 adapters removed were trimmed of low-quality reads using Trimmomatic program^27^ for further analysis. The degree of viral enrichment and non-viral contamination were investigated using the ViromeQC program^28^ with -w environmental option. Within quality trimmed metagenome reads, the enrichment scores were computed by dividing the abundance of bacterial 16 S small subunit ribosomal RNA gene (SSU rRNA) and 23 S large subunit rRNA gene (LSU rRNA), as well as single-copy universal bacterial and archaeal marker gene sequences by those found within viromes.

The trimmed reads were assembled using SPAdes version 3.5.0 (for ’14 Oct. and ’15 Jan. samples) and 3.8.2 (for all the other samples)^29^ with k-mer values of 27, 47, 67, 87, 107, and 127 and–careful option. Of all the constructed contigs, only those that were 10-kb in length or longer were selected for VirSorter analysis (Table 2). All the selected contigs from Lake Soyang were used as an input to VirSorter algorithm^22^ with the virome decontamination option using the virome database to screen for contigs that are of the viral or prophage origin (http://de.cyverse.org/de/). The VirSorter identified viral or prophage contigs by searching for viral proteins within the submitted contigs. Based on the number of viral protein-coding genes found, the submitted contigs were classified into three categories, “pretty sure,” “quite sure,” and “not so sure.” For further analysis, only the contigs that were classified as “pretty sure” and “quite sure” categories were accepted (Table 2)^24^.

## Data Records

The raw data of Lake Soyang viromes are available on the European Nucleotide Archive (ENA) under the accession number of PRJEB15535 (ERP017347)^30^. The virome reads from which PhiX174 and adapters had been removed were uploaded in the MG-RAST server for basic analysis under the accession numbers of mgm4632933.3 (’14 Oct.), mgm4632937.3 (’15 Jan.), mgm4694059.3 (’15 Sept.), mgm4709782.3 (’15 Nov.), mgm4709783.3 (’16 Feb.), and mgm4709863.3 (’16 May)^31^. The virome contigs that were 10-kb in length or longer were selected and deposited in the JGI IMG/MER database with accession numbers of IMG3300007735 (’14 Oct.), IMG3300007734 (’15 Jan.), IMG3300011113 (’15 Sept.), IMG3300011116 (’15 Nov.), IMG3300011114 (’16 Feb.), and IMG3300011115 (’16 May)^32^.

## Technical Validation

The virome reads were evaluated for their sequencing qualities using the fastp program^33^, using default parameters. The Q scores for the raw virome reads were calculated and showed that 68.35 to 73.78% of reads scored Q30 or higher (Table 4), indicating that most of the virome reads were constructed with low error rates. To evaluate how well the purification protocol employed in this study enriched virus-like particles (VLPs) and reduced contamination by bacteria, ViromeQC program was used. For each virome, ViromeQC first calculates the proportion of virome reads that are aligned to SSU and LSU rRNA gene sequences obtained from the Silva database, or matched to 31 conserved bacterial marker proteins database. Then, this program calculates enrichment scores by dividing the median proportions calculated from >2,000 non-enriched (i.e. non-viral) metagenomes by the proportions calculated form each virome, with the underlying premise that better enrichment of VLPs would lead to the decrease of aligned or matched reads, resulting in the increase of enrichment score. Among the three enrichment scores (SSU rRNA, LSU rRNA, and marker proteins), the minimum one is regarded as a comprehensive enrichment score. The enrichment score of Lake Soyang viromes ranged from 2.95 to 8.29 (median: 6.56), which indicates that the purification protocol of this study worked well compared to the scores of ~2,000 viromes calculated by ViromeQC where ~50% of viromes showed enrichment scores of ≤3^28^.

**Table 4:** The Q scores of raw virome read collected from Lake Soyang.

Sample	Base pair (Gbp)	Q20 (Gbp)	Q20 (%)	Q30 (Gbp)	Q30 (%)	GC content (%)
′14 Oct.	3.20	2.67	83.45	2.28	71.00	48.38
′15 Jan.	3.08	2.52	81.64	2.12	68.64	49.24
′15 Sept.	5.54	4.53	81.82	3.82	68.99	44.71
′15 Nov.	3.69	3.10	83.94	2.72	73.78	46.85
′16 Feb.	3.25	2.68	82.60	2.34	72.18	49.04
′16 May	3.18	2.63	82.88	2.31	72.53	47.96

## Data Availability

The options used for the generation and processing of the virome data are as follows: Trimmomatic (v. 0.33): ILLUMINACLIP: TruSeq. 3-PE-2.fa:2:30:10 LEADING:10 TRAILING:10 SLIDINGWINDOW:4:16 MINLEN:100 SPAdes (v. 3.5.0 and v. 3.8.2): -k 27, 47, 67, 87, 107, 127--careful
